# Generation of vascular chimerism within donor organs

**DOI:** 10.1038/s41598-021-92823-7

**Published:** 2021-06-28

**Authors:** Shahar Cohen, Shirly Partouche, Michael Gurevich, Vladimir Tennak, Vadym Mezhybovsky, Dmitry Azarov, Sarit Soffer-Hirschberg, Benny Hovav, Hagit Niv-Drori, Chana Weiss, Adi Borovich, Guy Cohen, Avital Wertheimer, Golan Shukrun, Moshe Israeli, Vered Yahalom, Dorit Leshem-Lev, Leor Perl, Ran Kornowski, Arnon Wiznitzer, Ana Tobar, Meora Feinmesser, Eytan Mor, Eli Atar, Eviatar Nesher

**Affiliations:** 1 Laboratory for Organ Bioengineering, Rabin Medical Center, Petah Tikva, Israel; 2 Felsenstien Medical Research Center, Rabin Medical Center, Petah Tikva, Israel; 3 Department of Organ Transplantation, Rabin Medical Center, Petah Tikva, Israel; 4 Experimental Surgery Unit, Rabin Medical Center, Petah Tikva, Israel; 5 Department of Radiology, Rabin Medical Center, Petah Tikva, Israel; 6 Department of Pathology, Rabin Medical Center, Petah Tikva, Israel; 7 Helen Schneider Hospital for Women, Rabin Medical Center, Petah Tikva, Israel; 8 Sackler Faculty of Medicine, Tel Aviv University, Tel Aviv, Israel; 9 Division of Pediatric Cardiothoracic Surgery, Schneider Children’s Medical Center, Petah Tikva, Israel; 10 Department of Cardiothoracic Surgery, Rabin Medical Center, Petah Tikva, Israel; 11 Tissue Typing Laboratory, Rabin Medical Center, Petah Tikva, Israel; 12 Zefat Academic College, Zefat, Israel; 13 Blood Services and Apheresis Institute, Rabin Medical Center, Petah Tikva, Israel; 14 Department of Cardiology, Rabin Medical Center, Petah Tikva, Israel; 15 Transplantation Unit, Department of Surgery B, Sheba Medical Center, Ramat Gan, Israel

**Keywords:** Regenerative medicine, Tissue engineering, Translational research

## Abstract

Whole organ perfusion decellularization has been proposed as a promising method to generate non-immunogenic organs from allogeneic and xenogeneic donors. However, the ability to recellularize organ scaffolds with multiple patient-specific cells in a spatially controlled manner remains challenging. Here, we propose that replacing donor endothelial cells alone, while keeping the rest of the organ viable and functional, is more technically feasible, and may offer a significant shortcut in the efforts to engineer transplantable organs. Vascular decellularization was achieved ex vivo, under controlled machine perfusion conditions, in various rat and porcine organs, including the kidneys, liver, lungs, heart, aorta, hind limbs, and pancreas. In addition, vascular decellularization of selected organs was performed in situ, within the donor body, achieving better control over the perfusion process. Human placenta-derived endothelial progenitor cells (EPCs) were used as immunologically-acceptable human cells to repopulate the luminal surface of de-endothelialized aorta (in vitro), kidneys, lungs and hind limbs (ex vivo). This study provides evidence that artificially generating vascular chimerism is feasible and could potentially pave the way for crossing the immunological barrier to xenotransplantation, as well as reducing the immunological burden of allogeneic grafts.

## Introduction

The domestic pig has been proposed as a potential xenogeneic donor of organs for humans. Its availability, breeding characteristics and physiological similarities to human beings make it more favorable organ donor than non-human primates^1,2^.

Earlier attempts to transplant porcine organs in primates failed due to hyperacute rejection (HAR) that occurred minutes to hours after transplantation, mediated by natural antibodies against the α1,3-galactosyl galactose epitope (αGal) followed by complement activation^3^. With the emergence of advanced gene editing and cloning techniques, it is now possible to knockout major xeno-antigens such as the αGal^4^, and over-express various human transgenes to improve the immunological and coagulation compatibility between pigs and humans, potentially avoiding HAR^5,6^.

However, graft loss beyond the hyperacute phase still occurs. Such a delayed form of acute rejection occurs over period of days to weeks and is characterized by endothelial cell activation and the development of thrombotic microangiopathy, with platelet aggregation and destruction of the microvasculature^7,8^.

Donor endothelial cells play several key roles in transplant rejection, both as initiators, active participants, and targets of acute cellular- and antibody-mediated rejection^9^. Consequently, it has been suggested that direct targeting of the graft vasculature may reduce alloimmunity and prolong graft survival^10^. Previous efforts to modify the extent of endothelial activation were made in the form of immunocloaking the vasculature using a temporary barrier^11^ and silencing MHC antigen expression^12–14^.

Perfusion decellularization of whole organs, followed by recellularization with patient-derived cells, has been proposed as a promising method to generate non-immunogenic organs from allogeneic and xenogeneic donors^15^. This approach relies on complete removal of donor cells and antigens, leaving behind a non-functioning organ scaffold. Recellularization attempts face significant challenges, such as the availability of sufficient quantity of cells and cell types needed to rebuild a whole organ. In addition, the ability to recellularize cell-free scaffolds with multiple cell types in a spatially controlled manner remains challenging. In fact, to date, studies in pigs were mainly successful in demonstrating functional re-endothelialization of the vascular compartment of decellularized organs, adequate to maintain perfusion with recipient blood and prevent thrombosis^16–18^. While seeding with a second cell type (airway epithelial cells) enabled gas exchange with decellularized lungs^19^, graft functionality was severely limited due to lack of maturation and other missing cells types.

Based on the literature presented above, we hypothesized that replacing donor endothelial cells alone, while keeping the rest of the organ viable and functional, could be technically feasible, and may provide a significant shortcut in the efforts to engineer transplantable organs. We sought to use human placental ECPs as immunologically-acceptable human cell candidates to generate vascular chimerism within donor organs under controlled machine perfusion conditions, paving the way to future translation into clinical practice.

## Results

### Ex vivo perfusion of rat and porcine organs and decellularization of the vascular tree

**Figure 1:**
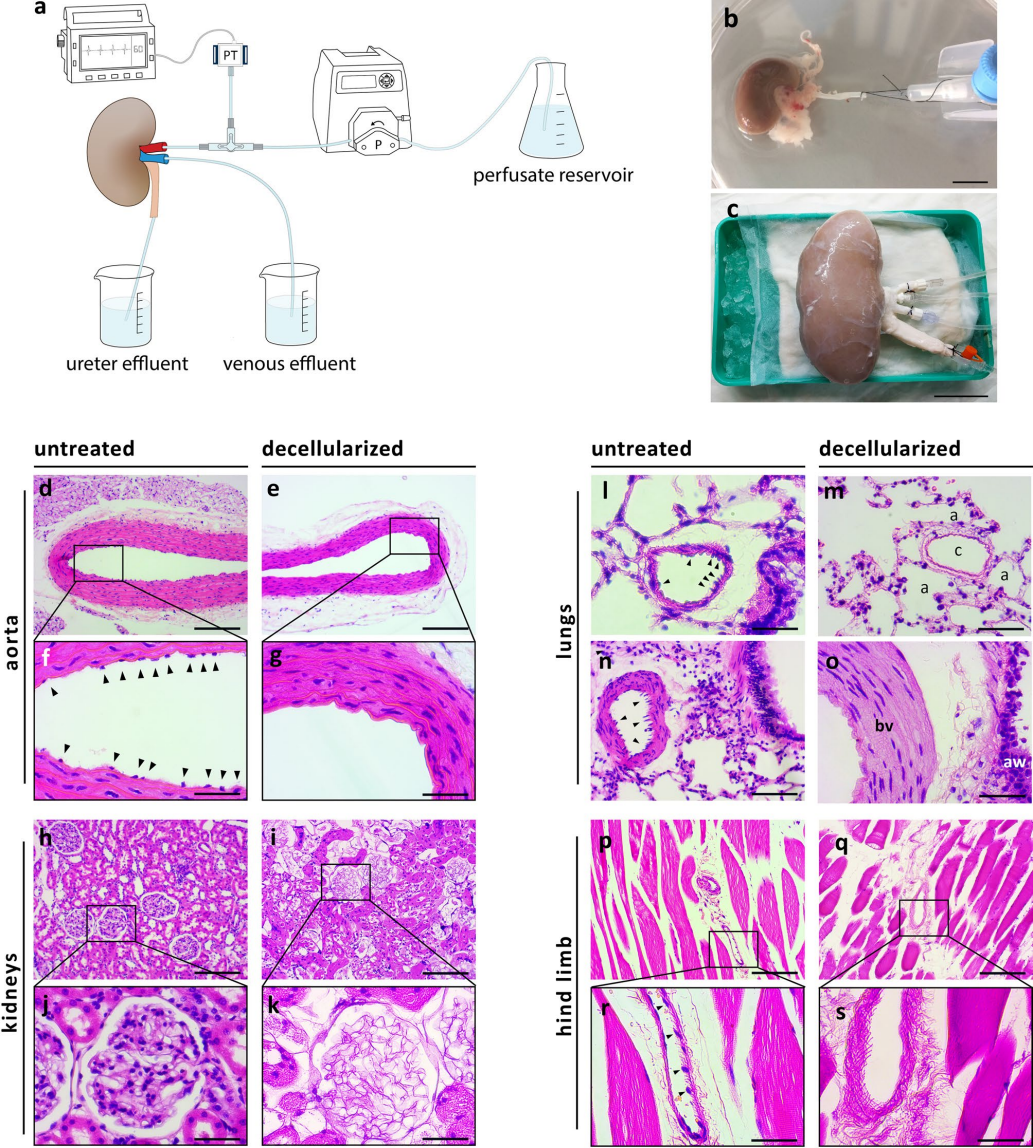
Ex vivo perfusion of rat and porcine organs and decellularization of the vascular tree. (**a**) Schematic representation of the hypothermic perfusion system used to decellularize rat and porcine organs. The circuit was driven by a peristaltic pump (Masterflex, Cole-Parmer) and controlled using a pressure transducer (Art-Line, Biometrix) and a monitor connected to the inflow cannula. (Illustration by Noa First Cohen). (**b**,**c**) Photographs of exemplary organs connected to the perfusion system: a rat kidney (**b**) cannulated using a 22G intravenous (IV) cannula placed in the abdominal aorta, and a porcine kidney (**c**) with Luer lock fittings placed into a single renal artery and two renal veins, and a 14G IV cannula placed into the ureter. Scale bars, 1 cm (B) and 5 cm (C). (**d**–**s**) Representative histological images of untreated and decellularized rat organs stained with hematoxylin and eosin (H&E): Low- and high-magnification of untreated aorta (**d**,**f**). Arrowheads show endothelial cells lining the luminal surface. Low- and high-magnification of decellularized aorta (**e**,**g**). Note the absence of the endothelial cell layer, while subendothelial tissue remains intact. Low- and high-magnification of untreated (**h**,**j**) and decellularized (**i**,**k**) rat kidneys. Note the appearance of acellular glomeruli with intact capillary wall structure, and preservation of epithelial cells of the surrounding collecting tubules. Representative images of untreated (**l**,**n**) and decellularized (**m**,**o**) lungs. Arrowheads show endothelial cells in untreated lung. Note the appearance of decellularized capillary surrounded by cellular alveoli (**m**) and de-endothelialized artery alongside cellular bronchiole (**o**). Low- and high-magnification of untreated (**p**,**r**) and decellularized (**q**,**s**) hind limb. Arrowheads show endothelial cells in untreated hind limb. PT, pressure transducer; P, pump; c, capillary; a, alveolus; bv, blood vessel; aw, airway. Scale bars, 50 µm (**f**,**g**,**j**,**k**,**l**,**o**,**r**,**s**), 100 µm (**m**,**n**) and 200 µm (**d**,**e**,**h**,**i**,**p**,**q**).

We first examined whether the vasculature of donor organs can be decellularized ex vivo, while preserving the remaining organ parenchyma viable and functional. Vascularized organs procured from rats and pigs were canulated and perfused with 4 °C 0.01–0.1% isotonic sodium dodecyl sulfate (SDS) at flow rates generating perfusion pressure of up to 120 mmHg (Fig. 1b,c). Upon initiating the SDS treatment, a temporary increase in perfusion pressure was detected, possibly due to increase in intraluminal cellular debris. Immediately following the SDS treatment organs were washed with saline, and perfusion pressures returned to baseline.

We were able to achieve a highly selective endothelial cell removal in the vascular tree of organs by perfusion with 0.1% SDS for a duration in the range of seconds (e.g. 20 s). Similar results were obtained by using lower SDS concentration (e.g. 0.025%) for longer exposure time (e.g. 20–30 min). However, this had implications on the efficiency of the washing process and consequently on the cold ischemic time: the higher SDS concentration used, the more intense the washing required to remove SDS remnants that might extravasate through the blood vessel wall and involve the surrounding tissue. The longer the washing phase, the longer the resulting cold ischemia time.

Choice of flow rates also had an effect on the efficiency of the decellularization treatment. Faster flow rates achieved more intense vascular treatment, that involved the entire vessel wall and the adjacent parenchyma. Faster flow rates also resulted in higher perfusion pressures and increase in movement of perfusate into the interstitial space, leading to organ edema.

Given the above, we tested several decellularization regimens for different organ types and sizes (Table 1).

**Table 1:** Selected decellularization regimens.

	Rat aorta	Rat lungs, hind limbs	Rat kidneys	Porcine kidney (I)	Porcine kidney (II)
SDS concentration	0.1%	0.025%	0.1%	0.05%	0.025%
Exposure time	5 s	20 min	10 s	3 min	20 min
Flow rate	1 mL/s	1–2 mL/min	1 mL/s	10–20 mL/min	20–40 mL/min

Histological examination demonstrated the removal of endothelial cells from the luminal surface at different levels of the vascular tree, including larger blood vessels (e.g. aorta, renal artery, renal vein, pulmonary artery and veins) and capillaries (e.g. kidney glomeruli and gas-exchanging capillaries in the lungs), with no detectable damage to the vascular basement membrane compared to untreated organs. Overall, the parenchymal tissue beyond the vascular wall was preserved (Fig. 1d–s and Supplementary Fig. S1). Trichrome, Elastic and PAS staining confirmed the preservation of collagen, elastin, and polysaccharides, respectively, all are key ECM components of the vascular wall (Supplementary Fig. S2). The removal of vascular endothelial cells was further confirmed in selected slides which stained negatively for CD31 and ERG, both endothelial cell markers (Supplementary Fig. S3).

### Human placental cell isolation and culture

Placental tissue fragments adhered to tissue culture plates were incubated uninterrupted until cells outgrowth began 5–7 days later.

**Figure 2:**
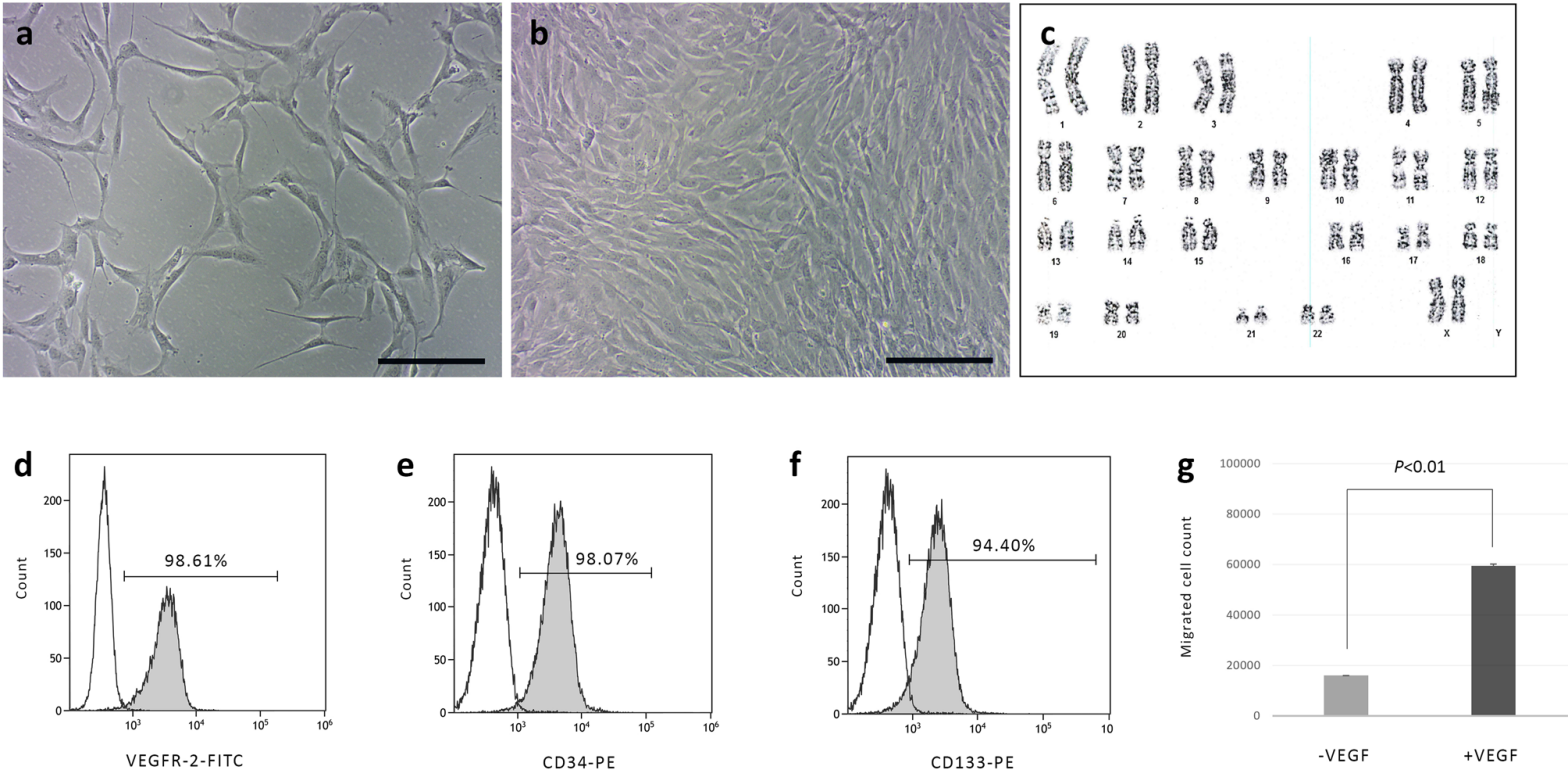
Isolation and characterization of human placental cells. (**a**,**b**) Microscopic images of human placental cells at low (**a**) and high (**b**) confluency. (**c**) Normal 46,XX karyotype of placental cells at passage 11. (**d**–**f**) Representative flow cytometry analysis, showing high expression level of EPC markers compared to unstained cells. (**g**) Placental cell migration in response to VEGF. Histogram is representative of cell counts in four random microscopic fields from two independent experiments (at passages 7 and 13). Bars indicate means ± SD (*P* < 0.01, non-parametric signed-rank test). Scale bars, 200 µm (**a**,**b**).

The isolated cells displayed spindle-shaped morphology, and aligned themselves to form capillary-like structures with a lumen within 6–12 h following routine trypsinization, a typical endothelial cell characteristic of angiogenesis, also used to identify EPCs^20^ (Fig. 2a). At 90–100% confluency the cells formed a more uniformed monolayer (Fig. 2b).

As previously shown^21^, cells isolated from different anatomical locations within the placenta displayed similar growth characteristics and phenotype (data not shown). In this study, only cells isolated from umbilical vein explants were further used.

Due to their marked proliferation capacity, karyotype analysis was performed at passage 11 revealing normal 46, XX karyotype with no chromosomal abnormalities (Fig. 2c).

Cell phenotype was analyzed by flow cytometry for the expression of CD34, CD133 and VEGFR-2, showing high expression levels at low and high passages (Fig. 2d–f). Migration assay demonstrated enhanced cell migration along a gradient of VEGF as a chemoattractant, further providing evidence for the cells angiogenic potential (Fig. 2g). Additional analysis of endothelia cell markers was performed by immunostaining for CD31, ERG and von Willebrand Factor (vWF), indicating the cells were positive for vWF but negative for CD31 and ERG (Supplementary Fig. 4).

Although ambiguity exists in the literature regarding the exact definition of an EPC phenotype^22,23^, our results indicated that the isolated cells could be termed placental EPCs.

### Cell seeding and generation of vascular chimerism in vitro

**Figure 3:**
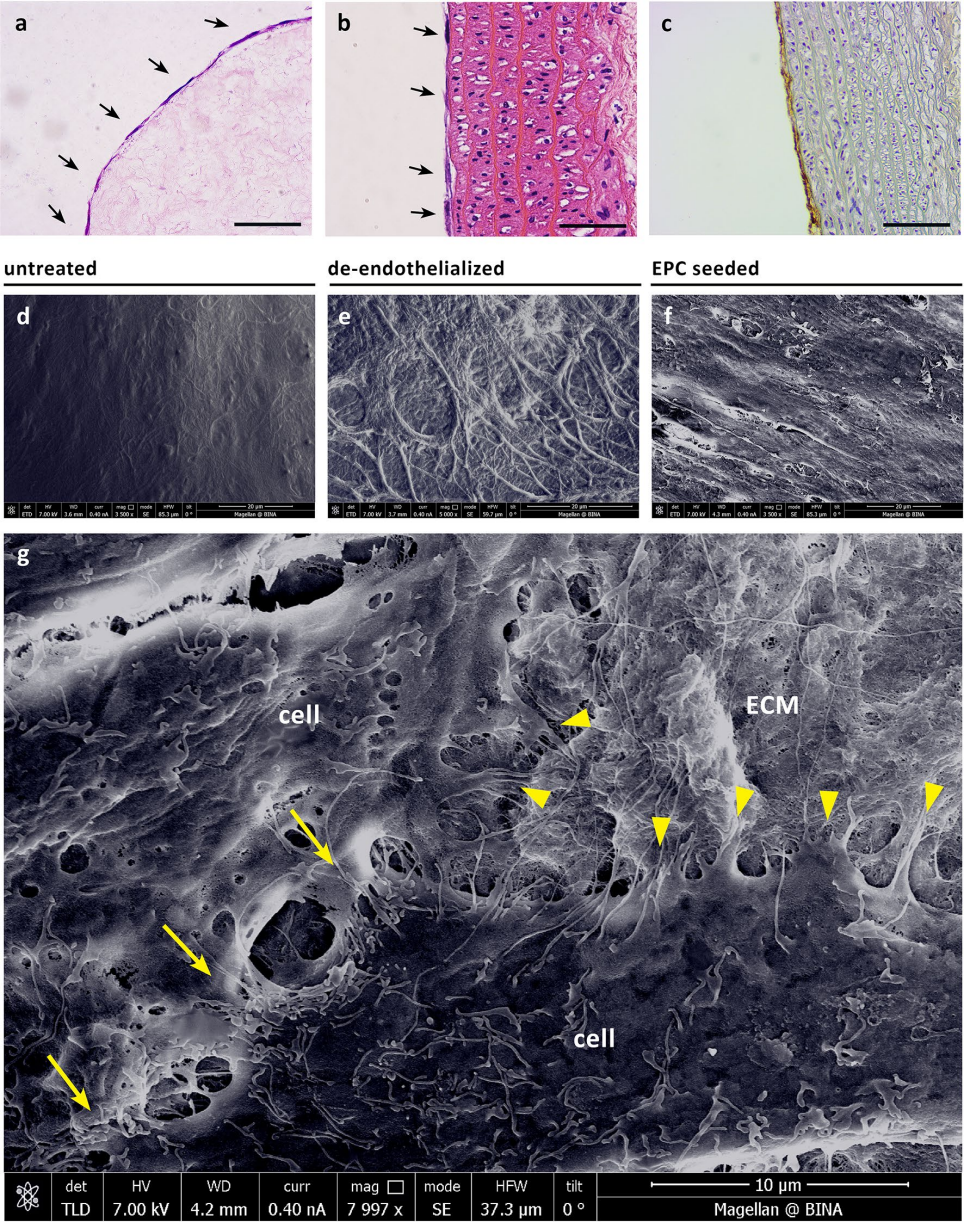
Cell seeding and generation of vascular chimerism in vitro. (**a**) H&E staining of human placental EPCs seeded on completely decellularized human umbilical vein. (**b**,**c**) H&E (B) and corresponding vWF immunohistochemistry staining (**c**) of human placental EPCs on de-endothelialized rat aorta. (**d**-**f**) SEM images of untreated (**d**), de-endothelialized and human EPC-seeded (**f**) rat aorta. (**g**) Corresponding high magnification SEM image of EPC-seeded aorta. Yellow arrows show cell-to-cell connections, yellow arrowheads show cell-to-ECM connections. Scale bars, 100 µm (**a**,**c**) and 50 µm (**b**).

Placental EPCs displayed high capacity to adhere to decellularized vascular surfaces. Initial seeding experiments using completely decellularized human umbilical veins demonstrated the ability of the cells to adopt flattened morphology and cover the luminal surface of the decellularized vein (Fig. 3a). We next examined the ability of the cells to repopulate segments of de-endothelialized rat aorta. Histological analysis of longitudinal sections demonstrated aligned orientation of placental EPCs in the original direction of the blood flow, within 5 days of static culture (Fig. 3b). Seeded cells were further identified by immunostaining for vWF (Fig. 3c). SEM imaging confirmed EPC alignment on de-endothelialized aorta (Fig. 3d–f). High magnification image revealed numerous cytoplasmic processes and intercellular connections indicative of active cell-to-cell and cell-to-ECM communication (Fig. 3g).

### Generation of vascular chimerism within rat organs

#### Isolated perfusion and vascular decellularization of rat organs in situ

**Figure 4:**
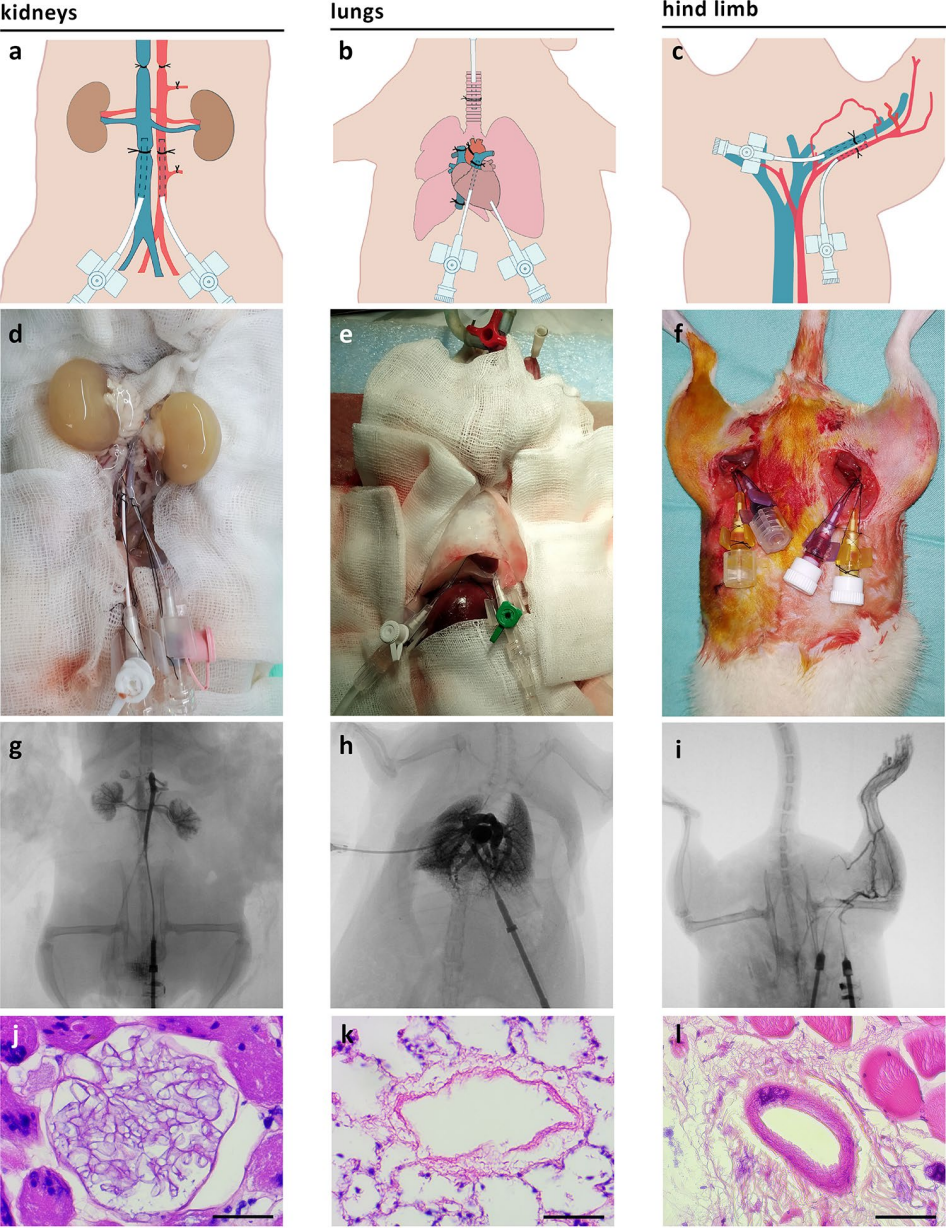
Isolated perfusion and vascular decellularization of rat organs in situ. Illustrations of isolated organ perfusion technique (**a–c**), corresponding photographs (**d–f**) angiographic images (**g–i**), and histological analysis (**j–l**) of rat kidneys, lungs, and hind limbs, respectively. Scale bars, 100 µm (**l**) and 50 µm (**j**,**k**). (Illustrations by Noa First Cohen).

By keeping the organ in its native anatomical location and performing vascular decellularization in situ, we were able to achieve more homogeneous perfusion, avoiding organ compression under gravity that could obstruct flow were the organ to be placed in its chamber. In addition, the in situ approach enabled better control over perfusate leakage, as fewer peri-organ dissections were required. Isolated perfusion of selected rat organs (kidneys, lungs, and hind limbs) was achieved by placing inflow and outflow cannulas into organ-specific locations, and ligating other vessels to prevent undesired flow, as depicted in Fig. 4. We used fluoroscopic angiography to visualize the isolated organs in situ. We recently suggested that this imaging modality should be routinely used for assessing vascular integrity in bioengineered organs^24^. Successful isolated perfusion was confirmed by fluoroscopic angiography (Fig. 4g–i and Supplementary Movies S1–S3), showing contrast medium passing through the organ arterial system directly to the venous system, with neglectable flow though side branches and collateral vessels.

#### Normothermic perfusion, cell seeding and generation of vascular chimerism ex vivo

**Figure 5:**
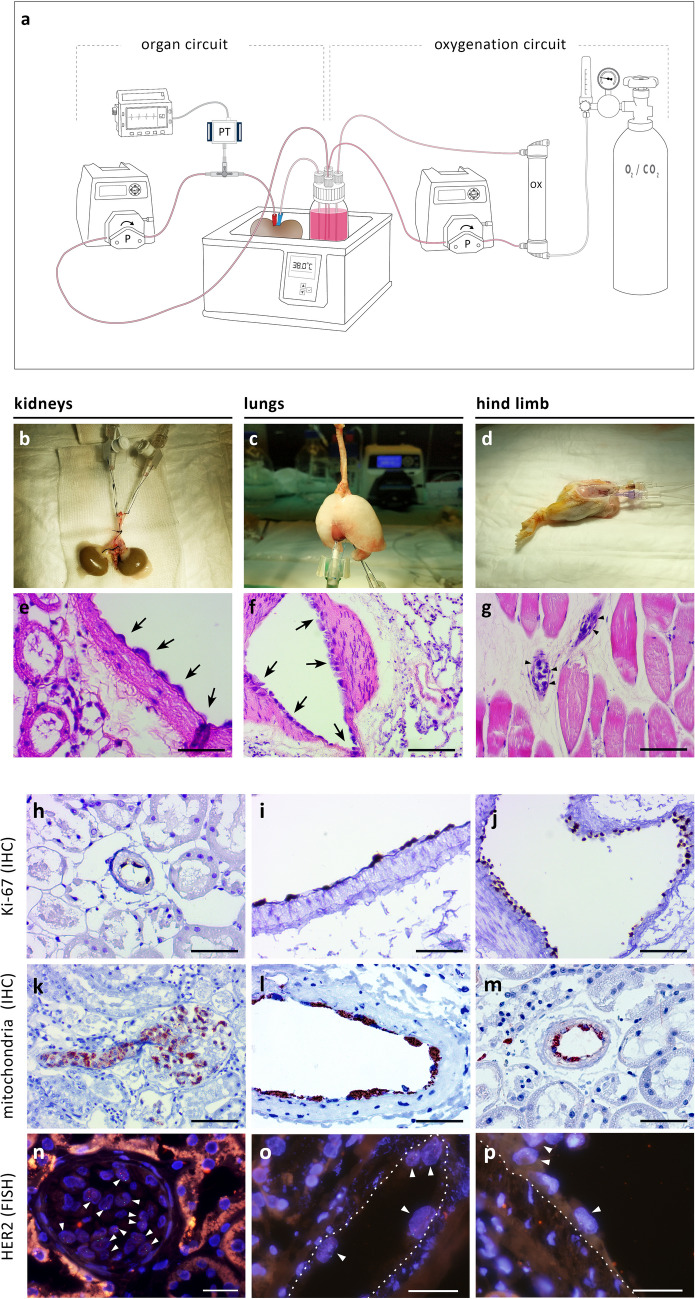
Normothermic perfusion, cell seeding and generation of vascular chimerism ex vivo. (**a**) Schematic representation of the normothermic perfusion system setup. The organ circuit was driven in a similar way to the hypothermic perfusion system described in Fig. 1a. The oxygenation circuit included a dialyzer used as membrane oxygenator (FX-10, Fresenius Medical Care) that was ventilated with 95% O_2_/5% CO_2_. Both the organ and the perfusate reservoir were placed in a heated water bath. (Illustration by Noa First Cohen). (**b**–**d**) Photographs of procured rat kidneys (**b**), lungs (**c**) and hind limb (**d**) together with their inflow and outflow cannulas, perfused ex vivo. (**e**–**g**) Corresponding H&E histological images. Arrows show human ECPs lining de-endothelialized rat blood vessels. (**h**–**p)** Seeded cells were further identified by immunostaining with anti-human mitochondria and anti-Ki67 antibodies, and FISH with DNA probe against human HER-2. Representative images showing Ki67 staining in kidneys (**h**) and lungs (**i**,**j**), human mitochondria staining in kidneys (**k**–**m**), and HER-2 staining in kidneys (**n**–**p**). Note positively-stained human cells rat blood vessels, alongside negatively-stained rat cells, generating vascular chimerism. White arrowheads show HER-2 positive cells displaying orange and green fluorescent dot signals inside cell nuclei stained with DAPI (blue). Dotted lines differentiate between positively-stained human EPCs and subendothelial tissue. Scale bars, 50 µm (**e**,**h**,**i**,**l**,**m**), 100 µm (**f**,**g**,**j**,**k**) and 25 µm (**n**–**p**).

We procured rat kidneys, lungs and hind limbs (Fig. 5b–d) and perfused them with oxygenated growth medium at 37 °C to achieve pH of 7.4 , *P*CO_2_ of 30–35 mmHg and *P*O_2_ of > 300 mmHg.

We used CD34+, CD133+, VEGF-R2 + human, placenta-derived EPCs to repopulate the vasculature of rat organs. A total of 4 × 10^7^ EPCs were seeded through the inflow cannula in each organ, and cultured under normothermic perfusion conditions for 4 h. Histological analysis of representative sections showed evidence of EPC engraftment in various size blood vessels (Fig. 5e–g). Human cells were clearly distinguishable from rat cells by their larger cell nuclei. To further confirm the human identity of the seeded cells, we utilized two methodologies: immunostaining with antibodies against ki67 antigen (Fig. 5h–j) and human mitochondria (Fig. 5k–m), and FISH with DNA probe against human HER-2 (Fig. 5n–p). Control slides confirmed that both the anti-mitochondria antibody and the HER-2 probe were human-specific. Anti-Ki67 antibody was used to differentiate between proliferating human EPCs, and quiescent, negatively-stained rat cells. We were able to visualize positively-stained human EPCs lining the luminal surface of rat blood vessels, alongside negatively-stained rat cells within the tunica media and beyond, artificially generating vascular chimerism. Occasionally, areas of suboptimal cell distribution were observed, with either under- or overseeded vessels clogged with cells (Supplementary Fig. S5).

For the duration of the 4-h culture period, perfusion pressures (representing pressure in the arterial system) were maintained stable. While venous outflow was drained back to the medium reservoir, some medium was also detected leaking into the organ chamber (Supplementary Fig. S6), probably originating from undetectable open branches of the venous system, as well as from microvasculature of dissected connective tissue adjacent to the organ. With amputated hind limbs, perfusate leaked from the open wound surface into the organ chamber.

## Discussion

The shortage of transplantable organs remains a global, unmet problem. Moreover, transplant waiting lists under-represent the true number of people who could benefit from a new organ. For example, it has been estimated that the true need for heart transplants in the US is more than ten times the heart transplant waiting list^25^. Those patients who do get an organ, must adhere to lifelong immunosuppressive therapy which expose them to increased risk of serious infection and cancer. In addition, organ transplants may still fail due to chronic rejection, leading to 5-year graft survival rate of around 80% (for kidneys)^26^ and 50% (for lungs)^27^.

Endothelial chimerism occurs naturally in organ transplants and more frequently when vascular injury occurs^28–32^. It has been demonstrated that recipient bone-marrow derived cells can replace donor endothelial cells and that this process is linked to immune tolerance^33,34^. Chimerism can extend beyond the endothelial cell layer and involve the entire blood vessel wall. Cells of host origin may replace graft vascular smooth muscle cells during vascular repair process^30^.

In contrast with solid, vascularized organs, non-vascularized grafts such as pancreatic islets are revascularized by blood vessels that grow predominantly from recipient endothelial cells via angiogenesis. It has been shown that following islet transplantation in mice, a progressive replacement of donor endothelial cells by the endothelial cells of recipient origin occurs, and that this type of endothelial chimerism play a role in protecting the islet grafts from humoral rejection^35^. This is further supported by earlier reports, describing the ability of avascular, embryonic kidneys^36,37^ and pancreas^38,39^ to attract host vasculature, rendering them less susceptible to humoral rejection^40^.

Based on this, and given the key role that donor endothelial cells play in graft rejection, we believe that artificially generating vascular chimerism could render donor organs more immunologically acceptable. In addition, from a pure tissue engineering perspective, the organ’s blood vessels form the interface between the donor and the recipient. As long as the organ survives, the recipient’s immune system comes in direct and continuous contact with its endothelial layer. Replacing this layer by means of tissue engineering could pave the way to immune tolerance.

In a recent report, human-pig chimeric embryos were made by inserting genetically modified human induced pluripotent stem cells (iPSCs, overexpressing an antiapoptotic factor) into pig embryos engineered using gene editing and somatic nuclear transfer^41^. In these chimeric embryos, all endothelial cells were of human origin. However, as discussed by the authors, safety, efficiency and ethical concerns need to be addressed before this technology can become clinically applicable^42^.

Here, we used ex vivo perfusion techniques in order to first selectively decellularize donor organ vasculature and then repopulate it with immunologically-acceptable human cells, thus artificially generating vascular chimerism.

Current decellularization approaches utilize intense detergent treatment and are challenged by the need to balance between the need for complete cell removal with minimal disruption of ECM structure and function (caused by detergents)^43^. Furthermore, it has been shown that residual SDS may have a harmful, doze-dependent and exposure time-dependent effect on decellularized tissues^44^. In this study, we showed that efficient cell removal can be achieved with SDS at concentration as low as 0.01% and exposure time ranging between seconds to minutes. In fact, at concentrations in the range 0.01% (corresponding to 100 parts per million) SDS is considered safe for use as a food additive according to the FDA’s Code of Federal Regulations Title 21 (CFR 172.822)^45^.

The extent of the decellularization dictates the type and number of cells needed for rebuilding the vasculature. We were able to achieve highly selective endothelial cell removal from large blood vessels. However, medium- and small-sized vessels were occasionally completely decellularized, with decellularization involving the surrounding tissue due to extravasation through the blood vessel wall. This warrants repopulation with cells that maintain phenotype plasticity, to be able to respond to local inductive ECM signals and transdifferentiate into cell type other than mature endothelial cells.

EPCs are circulating, bone marrow-derived cells characterized by their ability to proliferate and differentiate into mature, organ-specific endothelial cells^23^. However, EPCs can also be isolated from other tissues and organs, including the placenta^46^. From an immunological standpoint, the recipient’s own endothelial cells are the ideal choice for re-endothelialization of the vasculature of a graft. However, especially for older patients with complex medical history, such as organ failure, autologous, culture-expanded cells may not always be immediately available in sufficient quantity to repopulate an entire organ (and sometimes multiple organs)^15^. As a practical alternative to autologous cells, placental EPCs have a number of advantages. First, they can be obtained safely and non-invasively, and may be available on demand, in large quantity; second, mesenchymal cells derived from placental tissue possess unique immunomodulatory properties that may promote immune tolerance^47^, which is not surprising considering the key role the placenta plays in forming the immunological barrier that sustain maternal tolerance during pregnancy and thereby avoid rejection of the fetus^48,49^. To improve immunological compatibility between the placenta and the recipient, placental cells could be pre-screened and banked according to blood type and HLA, so that ABO- and HLA-compatible organs could be generated. Third, as recently shown, endothelial cells derived from human umbilical veins maintain some level of phenotypic plasticity, and therefore can differentiate into functional, organ-specific endothelium upon exposure to inductive, microenvironmental cues^18^.

In light of this, we used the human placenta to derive cells from umbilical vein explants. Our results show that the cell isolation and culture techniques employed in this study yielded endothelial progenitors, as the isolated cells were highly positive for CD34+/CD133+/VEGFR2+ (markers of early EPCs), negative for CD31 and ERG (markers of mature endothelial cells), and possessed angiogenic potential and marked proliferation capacity, while expanded under non-selective culture conditions. Seeding experiments demonstrated the cells’ ability to readily adhere to decellularized vascular surface in vitro, and engraft into vascularized organs ex vivo.

Based on the above, we propose that human placental EPCs could be used not merely as proof of concept for re-endothelialization studies, but also as a practical alternative for autologous endothelial cells, in order to humanize the vasculature of xenografts, as well as to reduce the immunological burden of allografts.

Furthermore, it has been shown that mesenchymal cells from various sources, including the placenta, exert their therapeutic effect through paracrine signaling, while having limited long-term engraftment and survival potential^47^. We hypothesize that this may in fact could be an advantage, and that human placental EPCs seeded in decellularized blood vessels could serve as temporary, trophic mediators, and progressively be replaced by recruited endothelial cells of recipient origin, post transplantation, generating stable, long-lasting chimerism.

The main limitation of our study is the nonoptimized seeding technique used to recellularize organs ex vivo. We used the same cell number, seeding volume and perfusion parameters for all organs regardless of organ type and size. The normothermic perfusion phase was limited to 4 h. Under these conditions, human EPCs were merely allowed to attach and begin to align, and in all organs sporadic areas of suboptimal cell distribution were observed. The goal in this study, though, was only to demonstrate the technical feasibility of using human placental EPCs for repopulation of the organ vasculature. Further optimization of seeding techniques and longer culture periods under physiological perfusion conditions are required to achieve homogenous distribution throughout the entire vascular tree and functional alignment of the seeded cells^15^.

Clearly, in order to obtain transplantable chimeric organs, functional endothelial barrier must first be restored. Endothelial cells play several roles in maintaining normal homeostasis, including regulation of blood pressure and tissue perfusion, regulation of gas and nutrient exchange, regulation of hemostasis and immune surveillance^10^. Several reports have demonstrated successful re-endothelialization of the vascular compartment in completely decellularized organs^17,18,50^. Endothelialization can be further enhanced by several means such as vascular surface modification using polymers with adhesive properties^51^, antibodies^16^, or growth factors^52^ to increase cell attachment, co-seeding with other cell types to support endothelial cell survival and function^53,54^, and changing organ posture during seeding to improve cell distribution throughout the organ vascular tree^55^, just to name a few. Remarkably, the inductive properties of decellularized ECM can induce phenotype change in seeded cells; that is, as long as the cells maintain phenotypic plasticity, they will differentiate and adopt tissue- and site-specific specialization^18,56^.

Whether full restoration of the endothelial layer is required prior to transplantation remains to be elucidated. It has been suggested before that the patient’s own body could be used as a living bioreactor for final maturation of engineered tissues^57^. In a more recent clinical report, acellular vessel conduits were repopulated by host progenitor and vascular cells post implantation, evolving over time into mature, multilayered, living blood vessels^58^. During that time, patients received antiplatelet alone or in combination with anticoagulants, to reduce the risk of thrombosis. It is tempting to speculate that in certain clinical scenarios, re-endothelialization and full restoration of the organ’s vasculature could be completed in vivo. To this extent, mobilizing agents such as granulocyte colony-stimulating factor (G-CSF) could be administered to increase the pool of circulating, bone marrow-derived EPCs and enhance their engraftment within the graft vasculature^59^. Presumably, there would be a latency period between transplantation and complete maturation of the organ, reflecting the time required for circulating EPCs of recipient origin to engraft, differentiate and regenerate the missing endothelium, during which immunogenicity and thrombogenicity would be increased. Optimization of immunosuppression and anticoagulation therapies would be required during that latency period, and by the end of it, the patient would be weaned off drug therapy, either partially or completely.

In this study we show that it is technically feasible to remove donor endothelium and replace it with immunologically-acceptable cells, and provide a strong rationale for pursuing such approach. Further studies are needed to confirm that the endothelial barrier is restored, to assess the functionality of the engineered organs (e.g. the ability to stimulate nerve and achieve muscle contraction in hindlimbs, the ability to produce urine in kidneys, the ability to achieve gas exchange in lungs), and to test hemocompatibility (via ex vivo perfusion with human blood).

We have also identified opportunities for future applications. Our proposed methodology could be applied more broadly to treat a variety of conditions associated with donor endothelial activation, disfunction or damage (for example, in acute settings resulting from ischemia/reperfusion injury, or in chronic conditions, leading to atherosclerosis^60^ or cancer^61^).

Greater understanding of the role placental EPCs may play in vascular regeneration and whether they could bridge the gap to a stable, recipient-donor chimerism, holds promise for developing xenogeneic and allogeneic grafts with blood vessels that may, over time, be rebuilt by the patient’s own cells.

**Figure 6:**
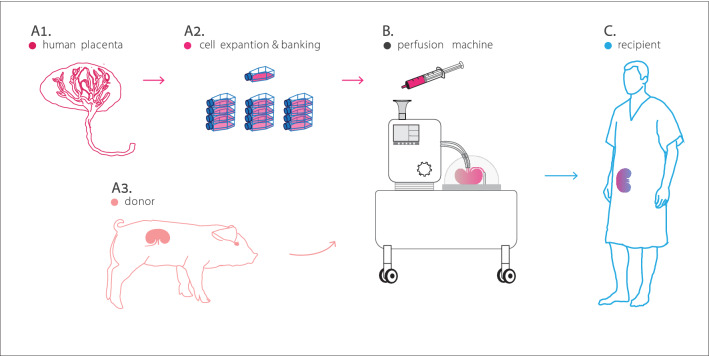
Suggested pathway to immune tolerance. An illustration demonstrating the proposed methodology for generation of transplantable, immunologically-acceptable organs for future translation into clinical practice. Donated human placenta (**A1**) are used to isolate, expand and bank ABO- and HLA-screened, immunologically-acceptable cells (**A2**). Pig organs (**A3**) are procured and mounted on a perfusion machine (**B**) used to rebuild the organ’s vasculature using ABO- and HLA-compatible placental cells. The immunologically-acceptable organ is transplanted into a recipient (**C**). (Illustration by Noa First Cohen).

Given the challenges associated with ongoing attempts to engineer transplantable pig organs by means of genetic engineering^62^ or whole organ perfusion decellularization/recellularization^63^, we believe that our approach (illustrated in Fig. 6) may provide a significant shortcut in the efforts to generate an unlimited supply of transplantable, immunologically-acceptable organs for future translation into clinical practice.

## Methods

All experimental protocols were approved by the Rabin Medical Center Institutional Animal Care and Use Committees (IACUC, permit no. 022-b-12639), and carried out in compliance with the Israeli Legislation and Regulation on the use of animals in biological and medical research with the ARRIVE guidelines.

Human placentae were donated with informed consent from women undergoing elective cesarean sections, and all the experimental protocols and the inform consent form were approved by the Rabin Medical Center Review Board (approval no. RMC-0547-20).

### Ex vivo perfusion of rat and porcine organs

Rat organs (kidneys, *n* = 8 pairs, liver, *n* = 3, lungs, *n* = 2, heart + lungs, *n* = 3, hind limb, *n* = 3, aorta, *n* = 10) were procured from Sprague Dawley female rats (300–500 g, Envigo, Israel) immediately following euthanasia. Organs were cannulated using 26-14G IV cannulas placed in the largest possible vessels (e.g. the abdominal aorta (AA) and inferior vena cava (IVC) for the kidneys, ilio-femoral vessels for the hind limb, portal vein, AA and IVC for the liver. Lungs were perfused through the pulmonary artery and the left ventricle of the heart).

Porcine organs (kidneys, *n* = 19, liver, *n* = 2, pancreas, *n* = 2, hind limb, *n* = 2) were procured from adult female domestic pigs (25–70 kg) immediately after euthanasia. Organ procurement was performed similarly as in clinical settings for human donors. At the back-table, Luer lock fittings were placed into the main vessels (e.g. renal artery, vein and ureter for the kidneys), and secured with silk 1–0 sutures.

All organs were initially flushed with 4 °C in 0.9% saline supplemented with 2 units/mL heparin (heparinized saline) at a hydrostatic pressure of 100 cm^2^ H_2_O, transferred to the lab on ice and stored at 4 °C in 0.9% saline supplemented with 1% antibiotics (penicillin, streptomycin and amphotericin B) until needed. Each organ was connected to a custom hypothermic perfusion system (Fig. 1a) and perfused with 4 °C heparinized saline.

### Decellularization of the vascular tree

Unlike previous decellularization attempts, where efforts are made to substantially remove all cellular material from an organ, here we only decellularized the vasculature, while keeping the rest of the cellular compartments of the organ viable and functional.

Endothelial cell removal was achieved by limited exposure of the organ vasculature to decellularization agents. 4 °C SDS in saline solutions were prepared at concentrations ranging between 0.01 to 0.1% and exposure times were adjusted empirically, ranging between seconds to minutes. Flow rates were applied to achieve perfusion pressure at physiological range, up to 120 mmHg. Additional optimization was made according to organ type and size. Selected decellularization regimens are shown in Table 1.

Following decellularization, organs were immediately perfused with 4 °C 0.9% saline on ice, under the same flow conditions for a duration of at least 10 times longer than the SDS perfusion step. After washing, tissue samples from each organ were fixed in 4% formaldehyde and processed for histological analysis.

### Human placental cell isolation and culture

Human placentae (*n* = 13) were obtained from full-term, normal pregnancies and transferred to the lab within 1 h postpartum. Tissue samples from different anatomical locations (chorionic villi, umbilical arteries, and umbilical vein) were rinsed in PBS to remove excess blood, and then dissected into 1–2 mm fragments and placed for adherence and cell outgrowth onto tissue culture plates. Isolated cells were cultured in the presence of alpha MEM supplemented with 15% FBS (growth medium) (both from Biological industries, Israel) at 37 °C and 5% CO_2_. Cells were passaged every 2–3 days at 90–100% confluency up to 23 passages (*n* = 2).

### Flow cytometry analysis

In order to identify endothelial progenitor cell (EPC) phenotype, placental cells at passages 12–23 (*n* = 5) were incubated with monoclonal antibodies against vascular endothelial growth factor receptor-2 (VEGFR-2, FITC labeled, R&D Systems), CD133 and CD34 (both PE-labeled, Miltenyi Biotech). Isotype-identical antibodies were used as controls. After incubation cells were washed with PBS and analyzed with a flow cytometer (FACSCalibur, BD Biosciences). Each analysis included 100,000 events, after selection for viability and exclusion of debris. In the next step, gated CD34 or CD133 positive cells were examined for the co-expression of VEGFR-2. Results were presented as the percentage of cells co-expressing either VEGFR-2 and CD133, or VEGFR-2 and CD34.

### Cell migration assay

In order to evaluate the isolated EPC angiogenic potential, a cell migration assay was performed. 10^5^ cells were placed in the upper part of a modified Boyden chamber. The chamber was placed in a 24-well culture dish containing growth medium and human recombinant VEGF (50 ng/mL) (PeproTech, Asia). After 24 h of incubation at 37 °C, the lower side of the filter was washed with PBS and fixed with 2% paraformaldehyde. For quantification, cell nuclei were stained with DAPI (Santa Cruz, CA, USA). Cells migrating into the lower chamber from were counted manually in four random microscopic fields.

### Karyotype analysis

In order to rule out chromosomal abnormalities in rapidly proliferating cells, karyotype of selected cell batch was analyzed at passage 12. Cell division was blocked at metaphase with 0.5 μg/mL colcemid (Biological Industries, Israel) for 1 h at 37 °C. The cells were washed and trypsinized, resuspended in 0.075 M KCl, incubated for 20 min at 37 °C, and fixed with methanol and acetic acid (3:1). G-band standard staining (Biological Industries, Israel) was used to visualize the chromosomes. 20 metaphase-nuclei were detected in each sample. The cells in metaphase were analyzed and reported on by a certified cytogenetic laboratory according to the International System for Human Cytogenetic Nomenclature.

### In vitro cell seeding experiments

To demonstrate the ability of human placental EPCs to re-endothelialize vascular surfaces, two types of matrices were prepared: completely decellularized human umbilical veins and de-endothelialized rat aorta.

Umbilical veins (*n* = 4) were cut into 5 mm^2^ samples, immersed in 0.1% SDS in PBS at room temperature overnight under constant agitation and then washed thoroughly with fresh PBS. Next, patches were glued onto tissue culture plates with luminal surface facing up and kept at 4 °C for up to 3 days until further use. Patches were incubated at 37 °C with growth medium 1 h before cell seeding. A total of 10^5^ placental EPCs at passages 1–4 were seeded per each patch and cultured with growth medium for 5 days.

Segments of AA (*n* = 6) were procured from rats immediately after euthanasia and placed on ice. 18-16G IV cannulas were secured on one side of the aorta and used for washing with 4 °C PBS. To selectively remove endothelial cells, 5 mL of 0.1% SDS in PBS were slowly injected, immediately followed by washing with fresh PBS. Washed aortic segments were cut longitudinally into rectangular shapes, glued onto tissue culture plates with luminal surface facing up, and incubated at 37 °C with growth medium 1 h before cell seeding. A total of 2 × 10^5^ placental EPCs at passages 2–8 were seeded per each patch and cultured with growth medium for 5 days.

At the end of the culture period, samples from each group were fixed in 4% formaldehyde, and processed for histology and immunohistochemistry analysis.

### Scanning electron microscopy (SEM) imaging of human EPC-seeded rat aorta

Visualization of native, de-endothelialized and EPC-seeded rat aorta samples was performed using scanning electron microscopy. Aortic patches (*n* = 2 from each group) were cut into 3 mm^2^ samples and immersed in Karnovsky's Fixative. Fixed samples were dehydrated and coated with iridium using a sputter coater (Q150R Plus, Quorum Technologies) and imaged on a Field-Emission Scanning Electron Microscope (Magellan 400L, FEI).

### Generation of vascular chimerism within rat organs

#### Isolated perfusion and vascular decellularization of rat organs in situ

In order to achieve homogeneous perfusion and better control over potential leakages that may result from peri-organ dissections during organ harvesting, an in situ isolated perfusion model was developed. Kidneys, lungs and hind limbs were chosen as representatives of abdominal organs, thoracic organs, and vascularized composite allografts (VCAs), respectively.

Sprague Dawley female rats (300–500 g) were anesthetized using inhaled isoflurane.

Isolated perfusion of the kidneys (*n* = 9 pairs of kidneys) was achieved by first placing an IV inflow cannula into the AA, and an outflow cannula into the IVC. Both cannulas were placed below the kidneys with their distal tip next to the root of the renal vessels. Systemic heparinization was then administered through the IVC. Above the kidneys, both the AA and IVC, as well as the portal vein, were ligated. Visible large arteries arising from the aorta such as the mesenteric and lumbar arteries were also ligated.

Isolated perfusion of the lungs (*n* = 9) was performed by first cannulating the trachea and initiating mechanical ventilation. An inflow cannula was placed through the right ventricle into the pulmonary artery (PA). An outflow cannula was placed in the left ventricle and secured using a purse-string suture. Systemic heparinization was then administered through the PA, and then the suprahepatic IVC and the ascending aorta were ligated.

Isolated perfusion of the hind limb (*n* = 12) was achieved by placing inflow and outflow IV cannulas into the femoral artery and vein, respectively. Systemic heparinization was administered through the inflow cannula. The counter hind limb femoral vessels were cannulated similarly to achieve isolated perfusion in both sides.

Following successful perfusion, animals were euthanized and placed on ice.

In order to achieve selective decellularization of the vascular tree, each cadaveric animal was connected to the hypothermic perfusion system (Fig. 1a) and the isolated organs were first washed with 4 °C heparinized saline to remove residual blood. Organ-specific decellularization regimens were applied (Table 1). Following decellularization, organs were perfused with 4 °C 0.9% saline under the same flow conditions for ten times longer than they had been during decellularization.

#### Visualization of perfused organs using fluoroscopic angiography in situ

In order to confirm isolated perfusion of the target organs, fluoroscopic angiography was performed with the Artis zee multi-purpose angiography system (Siemens Healthcare). 3–5 mL of Iopromide contrast medium (Ultravist 300, Bayer Healthcare) were administered into the inflow cannulas placed into the abdominal aorta, pulmonary artery and femoral artery for visualization of the kidneys, lungs and hind limb, respectively (*n* = 2 from each group).

#### Normothermic perfusion, cell seeding, and generation of vascular chimerism ex vivo

Each isolated organ (pair of kidneys, *n* = 8, lungs, *n* = 3, hind limb, *n* = 5) was procured together with its inflow and outflow cannulas and connected to a custom normothermic perfusion system (Fig. 5a).

In order to maintain perfusion under physiological conditions, growth medium alone was first heated to 38 °C (to compensate for heat loss from the tubing system) and circulated through the perfusion system for 1 h to achieve gas equilibrium. Samples were collected to determine pH, *P*O2 and *P*CO2 using an arterial blood gas analyzer (ABL835, Radiometer Medical ApS).

To achieve normothermic conditions, procured organs were first immersed in 37 °C saline in a heated water bath and then flushed with 37 °C equilibrated medium (eMedium).

In order to repopulate de-endothelialized rat organs with human cells, human placental EPCs were used. A total of 4 × 10^7^ EPCs at passage 3–15 were trypsinized, passed through a 70µ filter, resuspended in 2 mL eMedium and manually injected into the inflow cannula followed by 200 µL of fresh eMedium. Cells were allowed to attach for 1.5 h, after which perfusion was restarted at a flow rate of 2–4 mL/min. Repopulated organs were maintained at 37 °C and perfused with fresh eMedium for up to 4 h. The outflow cannula was allowed to drain passively into a medium reservoir during the culture period. At the end of the 4 h culture, organs were further flushed with 10 mL of fresh eMedium to remove unattached cells from the vasculature.

### Histological analysis

Tissue samples analyzed in this study were fixed in 4% formaldehyde for 24 h at room temperature, embedded in paraffin, cut into 5 µm-thick sections and stained with hematoxylin and eosin (H&E) for morphological examination and comparison. Hind limb samples were decalcified prior to paraffin embedding.

Selected sections were stained with trichrome (for collagen detection), PAS (for polysaccharides detection) and elastin staining kits according to the manufacture’s protocol.

For immunostaining of human mitochondria, deparaffinized sections were heated in sodium citrate buffer, pH 6.0, for 15 min and then cooled down to room temperature. Endogenous peroxidase was quenched in 3% hydrogen peroxide for 5 min in darkness, followed by rinsing in PBS. Slides were then blocked in 2.5% normal horse blocking serum (Vector Laboratories) at room temperature for 20 min, followed by overnight incubation at 4 °C with primary anti-mitochondria antibody (Abcam, ab-92824), diluted at 1:1000. Detection of primary antibody was performed with horseradish peroxidase-conjugated, horse anti-mouse, rat-adsorbed, secondary antibody (ImmPRESS, Vector Laboratories) and aminoethyl carbazole (AEC, Thermo Fisher Scientific) at room temperature for 30 min. Nuclei were counterstained with hematoxylin (Leica Biosystems). For immunostaining of Ki67, CD31, ERG and vWF, deparaffinized sections were pretreated with the ULTRA CC1 Solution for 64 min at 95 °C (for Ki67, CD31 and ERG) and protease-1 for 8 min at room temperature (for vWF) (both from Ventana Medical Systems), and incubated for 40 min at 37 °C with primary anti-Ki67 antibody (Thermo Fisher Scientific, RM-9106-S) diluted at 1:200, primary anti-CD31 antibody (M0823, Dako) diluted at 1:50, primary anti-ERG antibody (434R-16, Cell Marque) diluted at 1:100, and primary anti-vWF antibody (A0082, Dako) diluted at 1:300, respectively. Primary antibody detection was performed using the ultraView Universal DAB Detection Kit (Ventana Medical Systems) on the BenchMark ULTRA immunohistochemistry system (Roche Diagnostics). Control tissue slides and staining with secondary antibody only, were used to define appropriate antibody concentrations and confirm target specificity. Stained slides were observed under an inverted microscope (ECLIPSE Ts2, Nikon).

### Fluorescence in situ hybridization (FISH) analysis

FISH analysis was performed with a specific DNA probe for human HER-2 (PathVysion, Abbott). Paraffin embedded slides were deparaffinized, incubated with Tissue Pretreatment solution (SPoT-Light, Invitrogen) at 100 °C for 10 min, followed by protease digestion at 37 °C for 10 min. Next, slides were dehydrated and incubated with the HER-2 probe at 90 °C for 5 min for denaturation and then at 37 °C overnight for hybridization in a processing system (ThermoBrite, Abbott). Next, slides were washed with SSC/NP-40 buffer at 72 °C for 2 min, and air dried in darkness. Finally, nuclei were counterstained with diamidino-2-phenylindole dihydrochloride (DAPI). Slides were observed under a fluorescence microscope (Axio Imager Z2, Zeiss) at a 60× magnification with oil immersion objective. HER-2 positive cells displayed orange and green fluorescent signals.

### Statistical analysis

Data are shown as means ± standard deviation (SD). For the migration assay, a comparison between the two groups was performed using SAS (version 9.4, SAS Institute) and included the non-parametric signed-rank test, with *P* < 0.01 for significance.

## Supplementary Information

Supplementary Information.

Supplementary Video 1.

Supplementary Video 2.

Supplementary Video 3.

## Data Availability

All data generated or analyzed during this study are included in this published article and its Supplementary Information files.
