# Supplementary material for: Generation of vascular chimerism within donor organs
Source: Sci Rep. 2021 Jun 28;11:13437. doi: 10.1038/s41598-021-92823-7 (PMC8238957; doi:10.1038/s41598-021-92823-7)
Supplement: Supplementary file 1 — Supplementary Information. [file 41598_2021_92823_MOESM1_ESM.pdf]

Generation of vascular chimerism within donor organs

Shahar Cohen[1]*, Shirly Partouche[1,2], Michael Gurevich[3], Vladimir Tennak[3], Vadym Mezhybovsky[3], Dmitry Azarov[4], Sarit Soffer-Hirschberg[5], Benny Hovav[5], Hagit Niv-Drori[6], Chana Weiss[6], Adi Borovich[7,8], Guy Cohen[7], Avital Wertheimer[7,8], Golan Shukrun[9,10], Moshe Israeli[11,12], Vered Yahalom[13,8], Dorit Leshem-Lev[2,14], Leor Perl[8,14], Ran Kornowski[8,14], Arnon Wiznitzer[7,8], Ana Tobar[6,8], Meora Feinmesser[6,8], Eytan Mor[8,15**], Eli Atar[5,8**] and Eviatar Nesher[3**]

Supplementary Information:

Supplementary Figure S1.

Supplementary Figure S2.

Supplementary Figure S3.

Supplementary Figure S4.

Supplementary Figure S5.

Supplementary Figure S6.

Supplementary Movie S1.

Supplementary Movie S2.

Supplementary Movie S3.

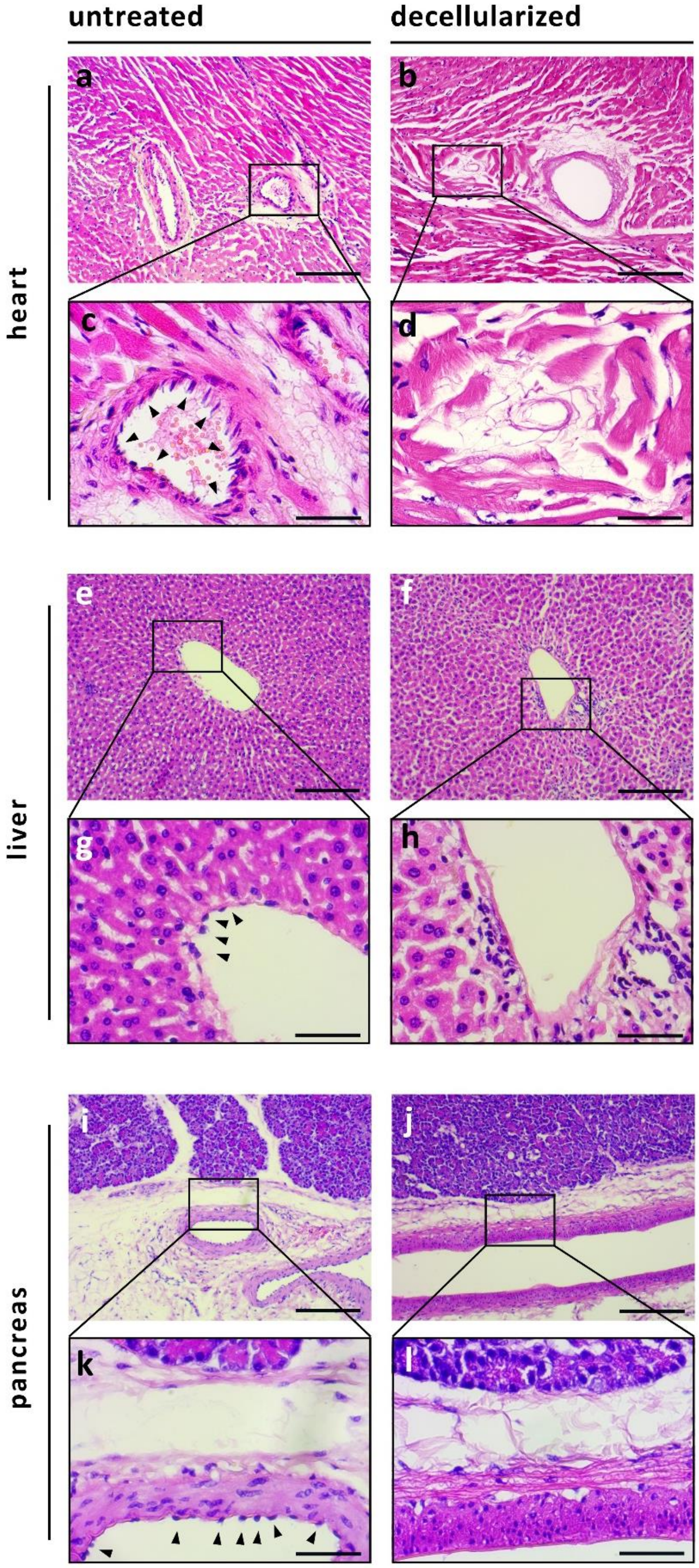

Supplementary Figure S1. ***Ex vivo*** **vascular decellularization of rat and porcine organs.** Representative low- and high-magnification H&E images of untreated and decellularized rat heart (**a**-**d**), liver (**e**-**h**) and porcine pancreas (**i**-**l**). Arrowheads show endothelial cells in untreated organs. Scale bars, 50 µm (c,d,g,h,k,l) and 200 µm (a,b,e,f,i,j).

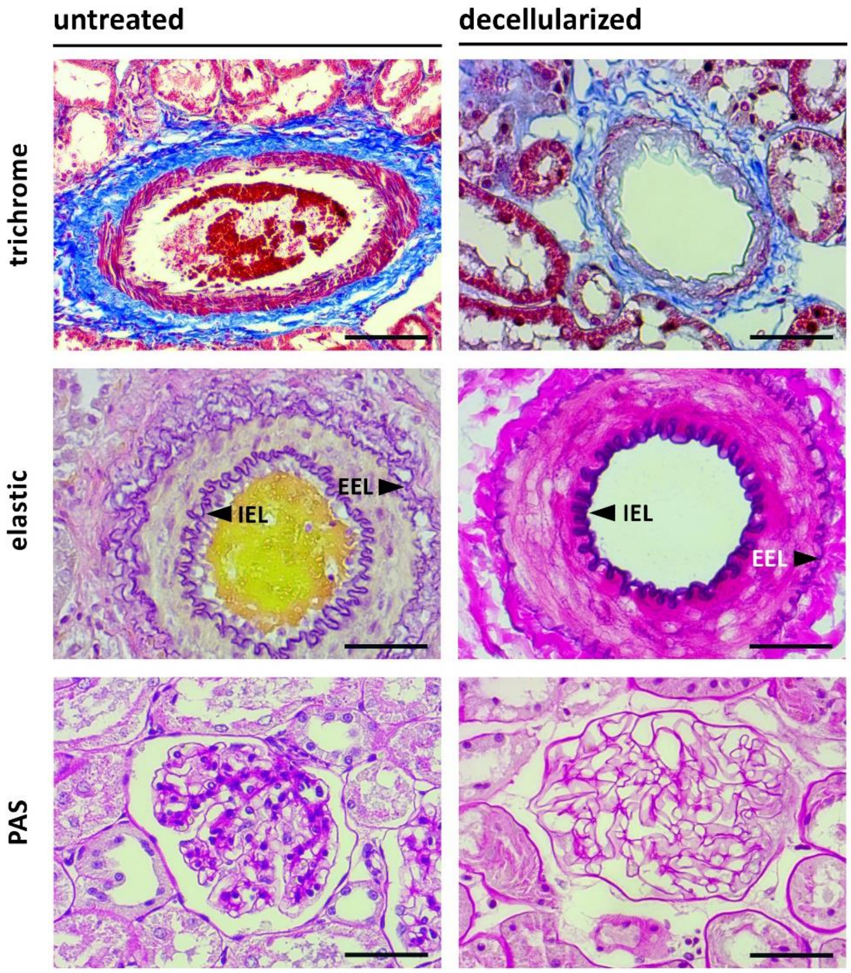

Supplementary Figure S2. Detection of key ECM components in decellularized kidneys. Representative histological images of untreated (left) and decellularized (right) rat kidneys stained with trichrome, elastic, and PAS staining, detecting collagen (blue), elastic fibers (dark blue-purple) and polysaccharides and basement membranes of glomerular capillaries (bright magenta), respectively. Arrows show internal elastic lamina (IEL) and external elastic lamina (EEL). Scale bars, 50 µm.

a

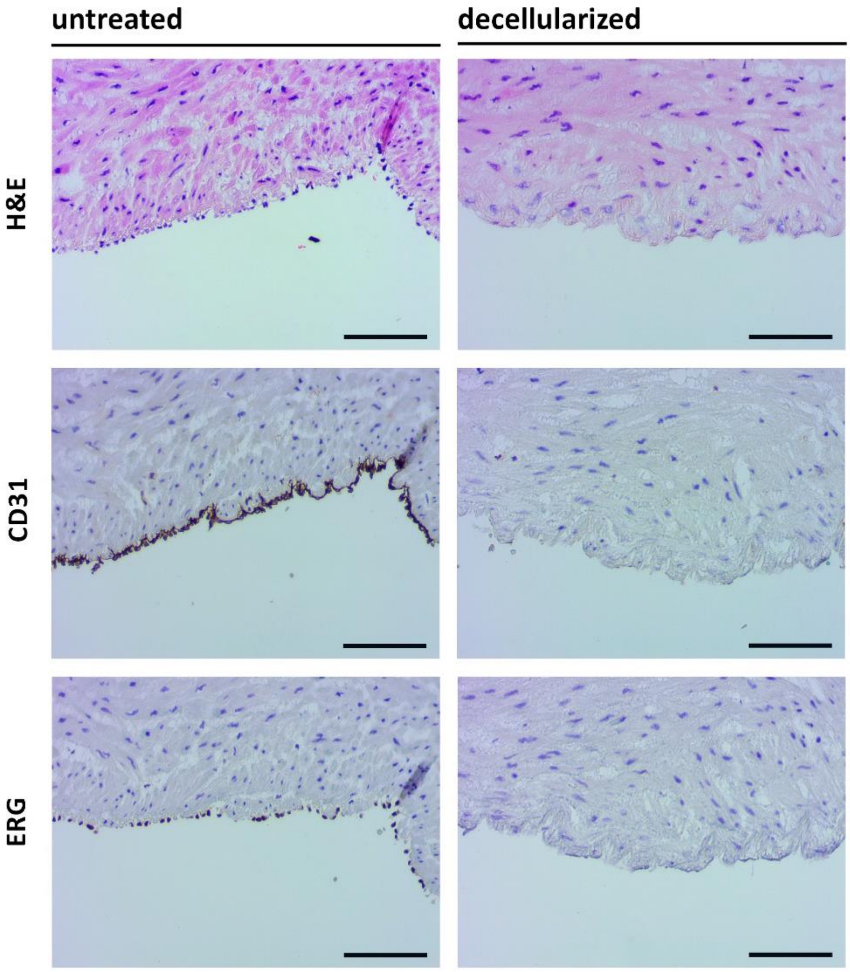

b

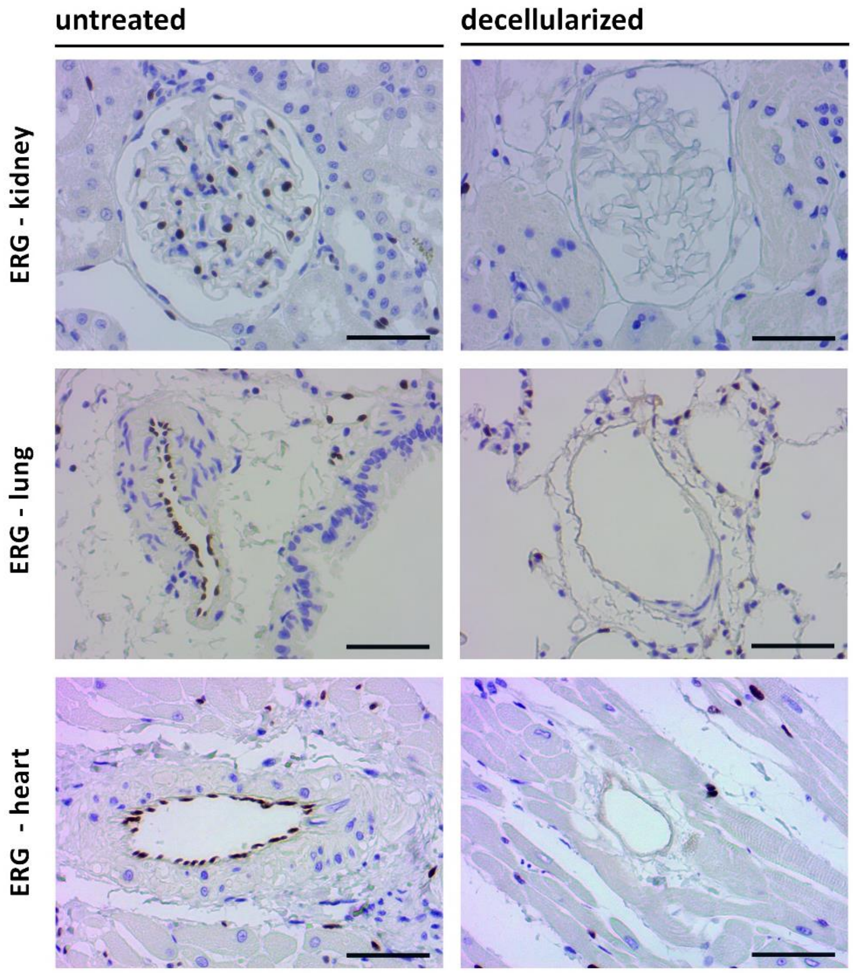

Supplementary Figure S3. Confirmation of endothelial cell removal from decellularized blood vessels. Representative H&E and corresponding immunohistochemistry images of untreated (left) and decellularized (right) human umbilical veins (**a**) and rat organs (**b**). Endothelial cells are identified by immunostaining with anti-CD31- and ERG antibodies. Scale bars, 100 µm (a) and 50 µm (b).

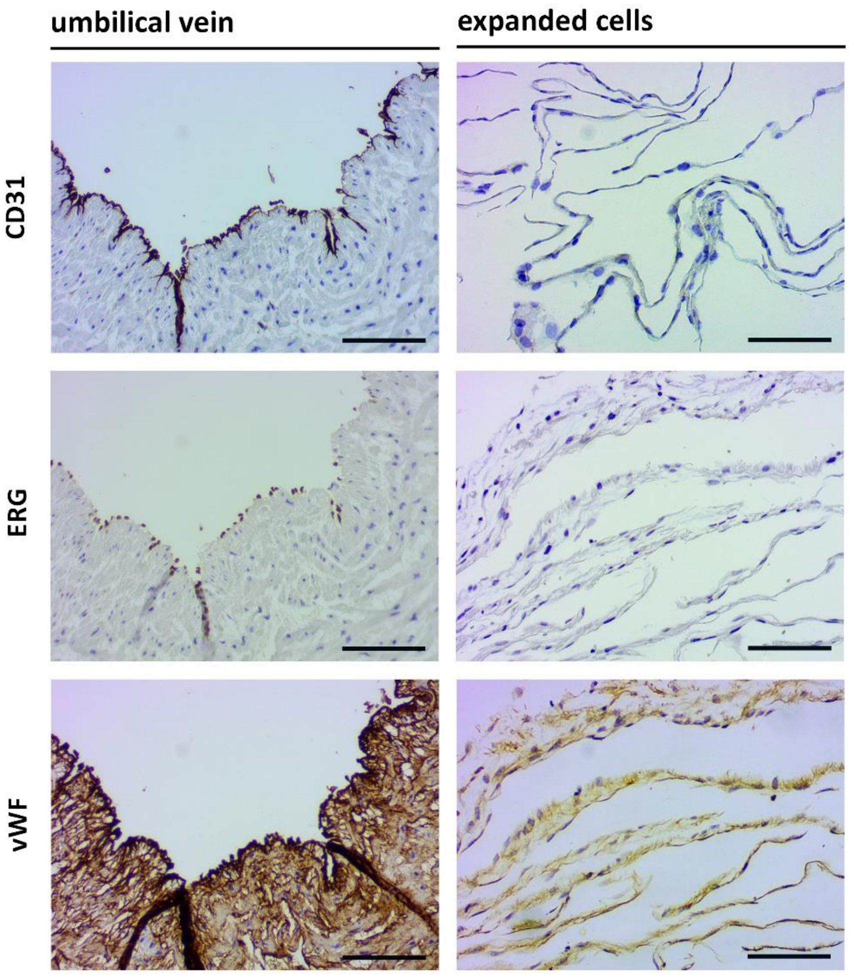

Supplementary Figure S4. Analysis of endothelial cell phenotype in placental cells. Representative immunohistochemistry images of placental cells expanded in culture and stained for CD31, ERG and vWF, to identify endothelial cell phenotype. Images are representative of confluent cultures of cells stained at passages 2, 4, 7 and 8. Expanded cells (right) were compared with positively stained control slides of umbilical veins (left). Scale bars, 100 µm.

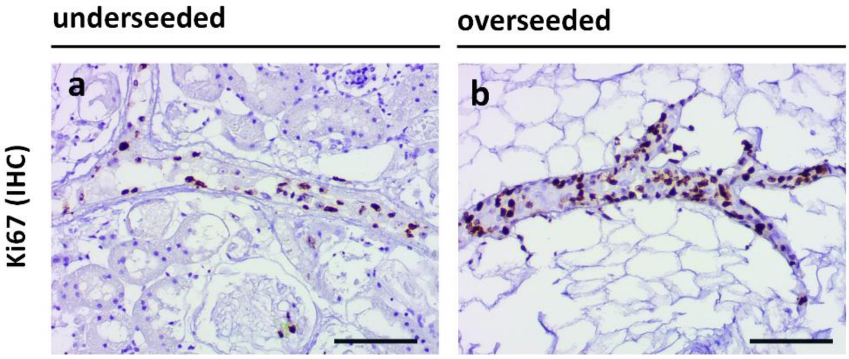

Supplementary Figure S5. Suboptimal cell distribution. Representative histological images of areas of suboptimal cell distribution within rat organs, showing underseeded kidney vessels (**a**) and overseeded, clogged lung vessels (**b**). Seeded human placental EPCs are identified by positive Ki67 immunostaining. Scale bars, 100 µm (a,b).

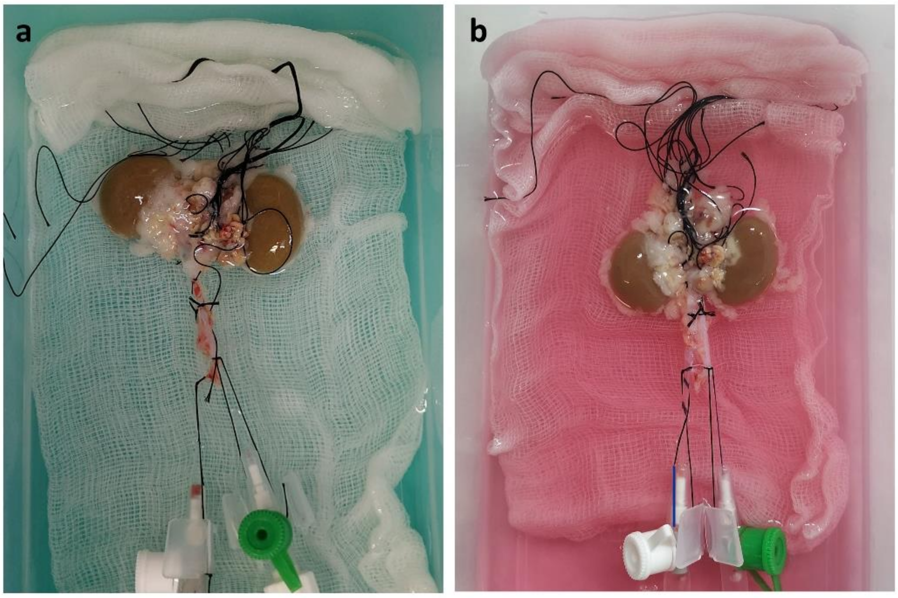

Supplementary Figure S6. Medium leakage into the organ chamber. Representative photographs of seeded rat kidneys placed inside the organ chamber, undergoing normothermic perfusion: leakage-free, at the beginning of the normothermic perfusion phase (**a**) and at a later stage, showing culture medium leaking into the organ chamber (**b**), presumably originating from undetectable open branches of the venous system, or microvasculature of dissected connective tissue adjacent to the kidneys.

Supplementary movies

Supplementary Movie S1. Representative fluoroscopic angiography confirming successful isolated perfusion of rat kidneys *in situ*.

Supplementary Movie S2. Representative fluoroscopic angiography confirming successful isolated perfusion of rat lungs *in situ*.

Supplementary Movie S3. Representative fluoroscopic angiography confirming successful isolated perfusion of rat hind limb *in situ*.
